# Association of social service counseling in breast cancer patients with financial problems, role functioning and employment—results from the prospective multicenter BRENDA II study

**DOI:** 10.1007/s00404-022-06604-2

**Published:** 2022-05-23

**Authors:** Davut Dayan, Elena Leinert, Susanne Singer, Wolfgang Janni, Thorsten Kühn, Felix Flock, Ricardo Felberbaum, Saskia-Laureen Herbert, Achim Wöckel, Lukas Schwentner, BRENDA-Study Group

**Affiliations:** 1 Department of Gynecology and Obstetrics, University of Ulm, Prittwitzstr. 43, 89075 Ulm, Germany; 2 Institute of Medical Biostatistics, Epidemiology and Informatics, University Medical Centre, Johannes Gutenberg University Mainz, Mainz, Germany; 3 Department of Gynecology and Obstetrics, Hospital Esslingen, Esslingen, Germany; 4 Department of Gynecology and Obstetrics, Hospital Memmingen, Memmingen, Germany; 5 Department of Gynecology and Obstetrics, Hospital Kempten, Kempten, Germany; 6 Department of Gynecology and Obstetrics, University of Würzburg, Würzburg, Germany

**Keywords:** Breast neoplasms, Financial problems, Problems with role functioning, Social service counseling

## Abstract

**Background:**

This study examined the relationship between social service counseling (SSC) and financial and role functioning problems in primary breast cancer (BC) patients over a 5-year observation period.

**Methods:**

In the multicenter prospective study, patients were approached before surgery (t1), before initiation of adjuvant treatment (t2), after therapy completion (t3), and 5 years after surgery (t4). We examined the proportion of BC survivors who had financial and role functioning problems and the proportion who were employed at t4. We examined how frequently patients were informed about, offered, or used SSC, and we used multivariate logistic regression analyses to examine the relationship between this and financial and role functioning problem prevalence.

**Results:**

Of the 456 BC survivors, 33% had financial problems and 22% reported role functioning problems at t4. There was no evidence that women with increased financial problems were informed about SSC more often than those without (OR 1.1, *p* = 0.84) or that they used SSC more often (OR 1.3, *p* = 0.25). However, women with role functioning problems were informed about SSC significantly more often (OR 1.7, *p* = 0.02) and attended counseling significantly more often (OR 1.6, *p* = 0.03). Among participants aged < 65 years at t4 (*n* = 255), 70% were employed. Patients who had received SSC were more likely to be employed at t4 than patients who did not (OR 1.9, *p* = 0.04).

**Conclusion:**

These findings underline the importance of SSC for BC patients with role functioning issues. They indicate that individuals who use SSC are more likely to be employed later on than individuals who do not.

## Introduction

Breast cancer (BC) is by far the most common type of cancer in women, with one in eight woman developing in the course of her lifetime. About one third of cases occur in women younger than 55 years [1] and, for them, successfully returning to work is often an important aim of supportive care and rehabilitation [2]. Technical advances in diagnostics and treatment have significantly improved disease prognosis [3]. The 10-year survival rate is 83%; this number is significantly higher when diagnosis occurs in the early stages of BC [1] and is steadily improving. In addition to medical consequences, a BC diagnosis also has psychological, social, professional and financial impacts on patients. On average, it takes patients 11.4 months between diagnosis and return to work [4–8]. Both the diagnosis and the side effects of treatment frequently affect the ability to work both physically and mentally, resulting in financial, social, and role function disadvantages. Numerous studies have investigated the effect of cancer on the quality of life of those affected, but with little consideration of financial aspects [9–11]. Financial well-being plays a very important role in patient quality of life. In their review, Smith et al. demonstrated that 49% of cancer patients suffer from financial distress related to treatment and chemotherapy side effects [12]. Jagsi et al. have shown that medical personnel responsible for treating patients sometimes only pay attention to acute treatment and do not notice other factors that are important for a healthy life after diagnosis [13]. It is important that, in addition to medical treatment of the disease, patients simultaneously receive support to manage various social impairments caused by the disease [14]. A questionnaire was developed by EORTC QLQ-C30 to determine where the threshold of clinically significant disease-related impairment of role functioning and financial problems lies [15].

### Aim

In German certified breast cancer centers, social service counseling (SSC) is mandatory and is an integral part of acute cancer treatment to support patients in reintegrating into everyday life. However, we lack data concerning the offer, attendance and benefits of SSC in adjuvant breast cancer care. Therefore, this prospective multicenter BRENDA II study examined the following research questions:

- Is SSC offered more frequently to patients with financial and role functioning problems? Do these groups participate more frequently in SSC compared to patients without financial or role functioning problems?
- Is employment higher in those who received SSC following adjuvant care?

## Methods

### Study design

This BRENDA II (Breast Cancer under Evidence-Based Guidelines) prospective multicenter cohort study evaluated patients with primary BC over a 4-year period (2009–2012). Patients were re-contacted and interviewed using the EORTC QLQ-C30 questionnaire at each visit (before surgery (t1), before initiation of adjuvant treatment (t2), after completion of adjuvant radio- and/or chemotherapy (t3), and again 5 years after surgery (t4)). In this study, patients with BC confirmed with primary histologic findings were included. Exclusion criteria were metastatic and/or recurrent disease, bilateral BC, primary occult disease, phylloides tumor, inability to complete a questionnaire, and no written informed consent for study participation. After a consultation, each patient was informed about the study by her physician and asked to participate. If she consented, the physician handed out the first set of questionnaires and interviewed the patient. Follow-up interviews were conducted by trained study nurses.

Data collection took place at four sites, all of which are BC centers certified by the German Cancer Society (Ulm University Hospital, Kempten Hospital, Memmingen Hospital, and Esslingen Hospital). Ethical approval was obtained from the ethics committee of the University of Ulm.

In breast cancer centers certified by the German Cancer Society, SSC must include identification of social, economic and psychological difficulties, planning and application for rehabilitation services, counselling patients in social and employment law, counselling patients in employment reintegration, and aiding patients with the application forms. SSC is conducted by specially trained social workers, and SSC in certified centers is controlled and audited regularly by the German Cancer Society.

### Instruments

Awareness and use of SSC was assessed in patients at t4 for a variety of services (inpatient and outpatient, e.g., hospital, inpatient rehabilitation clinic or cancer counseling services) using a questionnaire.

Financial problems were ascertained using the relevant subscale of the EORTC QLQ-C30 [15]. To indicate a group of patients with major financial difficulties, a threshold was used as defined in Giesinger et al. 2020 [16], i.e., a score of > 17 was considered to indicate elevated financial problems.

Role functioning (also captured with EORTC QLQ-C30) includes problems at work and during leisure time. Here, the threshold is 58. Since this is a functioning scale, the direction of scores is reversed and female patients with scores < 58 have suprathreshold problems in their role functioning at work and/or during leisure time.

Clinical data were obtained from medical records by trained data managers, and demographic data such as age, education, income, migration history, and employment status were self-reported by patients. Equivalent income was calculated using household net income divided by the number of adults and children in the household [17].

Participants who were born outside Germany and/or did not hold German citizenship were defined as having a "migration background".

### Statistical analysis

The proportion of patients with financial problems and problems in role functioning was computed stratified by educational level.

We calculated the percentage of SSC users in those with elevated problems versus those without.

Multivariate logistic regression analyses were performed to examine the relationship between offer and use of SSC and the existence of elevated financial and role functioning problems as exposure variables, while controlling for age, education, income, migration background, and risk group according St. Gallen criteria. Results are presented in odds ratios (OR) with 95% confidence intervals.

The relationship between use of social services and subsequent employment was also investigated using logistic regression. This analysis was restricted to patients of < 65 years of age and adjusted for employment at baseline, age, chemotherapy, and disease progression.

Statistical analyses were performed using STATA 15 (StataCorp 2017, College Station, TX: StataCorp LP).

## Results

### Sample

Of the 759 patients with primary breast cancer who participated at baseline, 60 had died by t4, 101 declined to participate again, 1 patient moved to an unknown address, and 141 could not be contacted, resulting in 456 participating survivors 5 years following diagnosis (60%). Participants were younger by an average of 7 years than dropouts and by an average 8 years than deceased patients. The clinical and demographic characteristics of the participating patients are shown in Table 1.

**Table 1:** Respondents’ demographic and clinical characteristics

	*N*	*%*
Age in years		
< 40	8	2
40–49	50	11
50–59	131	29
60–69	123	27
70–79	115	25
80 +	19	4
Unknown	10	2
Education in years		
< 10	195	43
≥ 10	256	56
Unknown	5	1
Current income in euros per person per month		
< 500	18	4
500–999	87	19
1000–1499	102	22
> 1500	186	41
Unknown	63	14
Immigrant		
No	387	85
Yes	64	14
Unknown	5	1
Locally advanced disease		
No	209	46
Yes	247	54
Surgical treatment		
Breast conserving	392	86
Mastectomy	64	14
Radiotherapy		
No	40	9
Yes	416	91
Chemotherapy		
No	247	54
Yes	209	46
Endocrine therapy		
No	84	18
Yes	372	82

### Frequency of financial problems and role functioning problems

Of participants at t4 (*n* = 456), 33% (*n* = 151) had relevant financial problems and 22% (*n* = 102) had role functioning problems. When results were stratified by education it was found that, for those with < 10 years of schooling, 35% (*n* = 69) had financial problems and 29% (*n* = 56) had role functioning problems (see Table 2).

**Table 2:** Financial impact and role functioning stratified for education

	Proportion worse than TCI			
	Financial impact		Role functioning	
	Yes	%	Yes	%
Education in years				
< 10	69	35	56	29
≥ 10	81	32	45	18
Unknown	1	20	1	20

### Offer and usage of SSC services 5 years after diagnosis in patients with and without financial and role functioning problems

Participants who reported financial problems during treatment (t2), were offered SSC (OR = 1.1, *p* = 0.84) and did not take advantage of SSC (OR = 1.3, *p* = 0.25) significantly more often than patients without financial problems.

However, participants with role functioning problems at t2 (adjusted for age, education, income, migration background and risk group according St. Gallen criteria) were offered SSC significantly more often (OR = 1.7, *p* = 0.02) and participated in SSC significantly more often (OR = 1.6, *p* = 0.03) compared to patients without role functioning problems (see Table 3).

**Table 3:** SSC offer and participation (adjusted for age, education, income, migration background and risk group according to St. Gallen)

*N* = *456*	Patients with or without financial Problems				Patients with or without role functioning problems			
	SSC offered		SSC used		SSC offered		SSC used	
	OR	*p*-value	OR	*p*-value	OR	*p*-value	OR	*p*-value
Below threshold	Reference							
Above threshold (i.e., with problems)	1.1	0.84	1.3	0.25	1.7	0.02	1.6	0.03
Unknown	1.1	0.74	1.2	0.53	1.6	0.15	1.4	0.31

### Employment 5 years after diagnosis, impact of SSC on employment rate

Of the 456 participants, 255 patients were < 65 years at t4, and about 70% were employed. The largest proportion of unemployed participants were housewives (12%), and 7% of participants were in early retirement. Only 1% of participants were unemployed (see Table 4).

**Table 4:** Employment among the participants < 65 years of age, 5 years after diagnosis

	*N*	%
Yes, fulltime	66	26
Yes, part time	83	33
Yes, less than part time	30	12
No, housewife	31	12
No, unemployed	3	1
No, disability retirement	11	4
No, retirement	19	7
No, other	11	4
Unknown	1	0.4

Study participants < 65y at t4 who participated in SSC during the course of their disease were significantly more likely to be employed 5 years after diagnosis than those who did not receive counseling (OR = 1.9, *p* = 0.04).

## Discussion

A diagnosis of cancer not only presents challenges during acute medical treatment but also has psychological, social, and financial consequences for affected women. Between 21% and 64% of cancer patients report financial difficulties [12, 18–24]. Financial burden results directly from cancer, treatment costs, and indirect costs (e.g., transportation costs, reduced productivity) [7, 12, 18, 20, 21, 25, 26]. Buettner et al. found that, despite the existing universal health care system, almost all female patients (97.8%) in Germany have to pay some kind of additional costs [18].

In this prospective study, we investigated the relationship between professional SSC among BC survivors in Germany and financial and role functioning problems. We found that 33% of all BC survivors had financial problems and 22% had role functioning problems. Lack of health insurance, lower income, unemployment [10, 12, 20, 22, 25], younger age [12, 13, 21, 23, 27, 28], nonwhite ethnicity, and chemotherapy treatment [13] are well-known risk factors. Other studies show that low financial status and lack of health insurance are associated with delay diagnosis and treatment [29–32]. Due to compulsory health insurance and universal health care in Germany, all patients have access to guideline adherent treatment. Finances are an important issue that clinicians rarely address. Some studies show that SSC enables better access to financial and social resources [33–35].

In our study, 70% of the participants were offered SSC; and only 9% of this group declined to take advantage of this offer. In their cross-sectional survey, Ko et al. found that, out of 77% of patients with social concerns, only 35% were offered SSC and only 20% received it [36]. In our study, twice as many patients were offered SSC and three times as many took advantage of it. This could be due to the fact that SSC is a mandatory offering at certified breast cancer centers in Germany. One reason for not seeking counseling, as described in other literature, may be participants’ unawareness of the benefits and advantages of counseling [33, 36, 37]. Jagsi et al. found that, out of members of the cancer treatment team, 50.9% of medical oncologists, 15.6% of surgeons, and 43.2% of radiation oncologists talk to patients about cancer-related financial burden. In contrast, the same paper reported that 72.8% of patients did not receive any assistance in this regard from the treating health personnel, and that 55.4% were not engaged in a discussion about this topic [13].

Ketterl et al. demonstrated that cancer treatment has a significant impact on physical and mental work ability and leisure activities among young cancer survivors (ages 18–39) [25]. Of the 84.4% participants employed at some time between cancer diagnosis and study participation (1–5 years post-diagnosis), 70.2% reported having an impairment and 58.6% reported a permanent impairment in physical abilities due to the disease and necessary treatment. Permanent mental impairment (to perform occupational activities) was reported by 54.2% of all cancer survivors [25]. In our study, 70% of patients who were younger than 65 years were employed 5 years after diagnosis. Ketterl et al. further demonstrated that, in comparison, employed participants with chemotherapy treatment were significantly more likely to have impairments in job-related mental tasks (OR = 2.66; *p* < 0.01) and were also more likely to take unpaid time off from work (*p* < 0.05). Those treated with radiotherapy were also significantly more likely to report impairment in mental tasks required for their work (*p* < 0.05) compared to those who did not receive radiotherapy [25].

In the Netherlands, Pearce et al. found that, despite the existing universal health care system, 22% of cancer survivors experienced financial hardship because of their physical condition or medical treatment. Those who were not employed had a higher risk of financial problems (27% vs. 16%, *p* < 0.001). They found that 49% of participants were employed. The Dutch and German health care system are similar thus comparable. In our study, 179 (70%, 255 participants < 65) participants were employed. However, it should be noted that only BC patients were included in our study, whereas the work of Pearce et al. included all types of cancer survivors (men and women) of whom only 12% had gynecologic cancers. Pearce et al. found 16% fewer cancer-related limitations in work or other activities in their study population compared with Ketterl et al. (54.2% vs. 42%) [21, 25]. Nonetheless, they reported that 35% of cases had changes in their work environment as a result of their cancer.

Participants who experienced problems regarding their role functioning during the course of the disease were more likely to be offered SSC within the first 5 years after diagnosis and to participate in SSC. Many cancer survivors seek a solution to their physical and mental alterations resulting from cancer diagnosis and treatment which affect their capacity at work. Welfare counselors have the legal and social expertise to improve access to supportive care and rehabilitation services according to the patients´ needs [33]. These findings are consistent with the literature and highlight the impact and need for social service.

Our study revealed that participants who received SSC were more likely to be employed at t4 than those who did not receive counseling. Our results are consistent with the literature [4, 5] and emphasize the need for SSC to support female cancer patients to reintegrate into daily life.

### Strengths and restrictions

The strength of this study lies in the prospective multicentric (all certified breast cancer centers) examination of the role of SSC in supporting patients in an area that has received very little research attention. This study focused on patient-centered views because they consider the entire course of the disease and do not just focus on the specific treatment period.

Out of 759 patients, we were only able to include 456 patients in the 5-year follow-up. We assessed the relationship between the SSC and financial and role functioning problems through a questionnaire. Like any self-reporting method, it could suffer from information bias and may not represent a measure of the actual impact on financial problems and role functioning.

## Conclusion

This prospective BRENDA-II study highlights the importance of SSC for BC patients with financial and role functioning problems. Individuals who receive SSC were more likely to be employed five years after surgery than individuals who did not receive counseling. SSC should be offered to all cancer patients to enable or facilitate support for integration into daily life and employment.
